# Cancer from a Clinical Standpoint, Some Peculiar Observations

**Published:** 1913-03

**Authors:** M. B. Hutchins

**Affiliations:** Atlanta, Georgia


### Journal-Record of Medicine

Successor to Atlanta Medical and Surgical Journal, Established 1855
and Southern Medical Record, Established 1870

**OWNED BY THE ATLANTA MEDICAL JOURNAL COMPANY**

PUBLISHED MONTHLY

*Official Organ Fulton County Medical Society, State Examining Board, Presbyterian Hospital, Atlanta, Birmingham and Atlantic Railroad Surgeons' Association, Chattahoochee Valley Medical and Surgical Association, Etc.*

EDGAR BALLENGER, M. D., Editor
BERNARD WOLFF, M. D., Supervising Editor
A. W. STIRLING, M. D., C. M., D. P. H.; J. S. HURT, B. Ph., M.D.
GEO. M. NILES, M. D., W. J. LOVE, M. D., (Ala.); Associate Editors
E. W. ALLEN, Business Manager

**COLLABORATORS**

DR. W. F. WESTMORLAND, General Surgery
F. W. McRAE, M. D., Abdominal Surgery
H. F. HARRIS, M. D., Pathology and Bacteriology
E. B. BLOCK, M. D., Diseases of the Nervous System
MICHAEL HOKE, M. D., Orthopedic Surgery
CYRUS W. STRICKLER, M. D., Legal Medicine and Medical Legislation
E. C. DAVIS, A. B., M. D., Obstetrics
E. G. JONES, A. B., M. D., Gynecology
R. T. DORSEY, Jr., B. S., M. D., Medicine
L. M. GAINES, A. B., M. D., Internal Medicine
GEO. C. MIZELL, M. D., Diseases of the Stomach and Intestines
L. B. CLARKE, M. D., Pediatrics
EDGAR PAULIN, M. D., Opsonic Medicine
THEODORE TOEPEL, M. D., Mechano Therapy
R. R. DALY, M. D., Medical Society
A. W. STIRLING, M. D., Etc., Diseases of the Eye, Ear, Nose and Throat
BERNARD WOLFF, M. D., Diseases of the Skin
E. G. BALLENGER, M. D., Diseases of the Genito-Urinary Organs

VOLUME LIX — MARCH, 1913 — No. 12

### CANCER FROM A CLINICAL STANDPOINT, SOME PECULIAR OBSERVATIONS

BY M. B. HUTCHINS, M. D. ATLANTA, GA

For several years a good deal of work has been done in the effort to learn the cause and cure of cancer, most of the experiments have been made with mouse cancer, but the disease in other mammalians, fish, birds, and even plants has been studied. Inoculations, chemical and serologic procedures reported would fill volumes. Results have been of little practical use so far, but we can hope for the privilege of living until this dreadful problem shall be solved and the present hopelessness be replaced by confidence that many more sufferers finally can be relieved. While waiting for this time it is well not to lose sight of clinical features and peculiarities in different forms of the disease, that may help fill the gaps in purely experimental work and aid in the solution of the problem.

Every accepted idea as to cancer could be contradicted by cases in my own experience and every usual observation has occurred. Heredity, contagion, auto-inoculation and even auto-suggestion of the disease has seemed demonstrated. On the other hand, every idea could be disproven by most of the cases. As to the popular blood theory—it is not worthy of consideration and especially if we have as corollary to it the futile use of blood medicines.

Harry Stillwell Edwards says there is no such thing as coincidence, every happening being the action of definite laws, or forces. Statistics prove things by the majority rule or by prejudice, and the cases I mention show simply what has happened, from which we can draw conclusions, or none, as our minds incline.

A woman of good health and heredity had a destructive abscess of the breast after the birth of her first child, over forty-five years ago. She is living, past seventy years of age. Her sister died of visceral metastasis from a little scirrhus of the right breast. Another sister married after having been a widow with one child for years; bore three or four children and died of cancer of the uterus. Both were robust women. The husband of the living sister came of a tubercular family, was about a third cousin of his wife. For years he lived in dread of cancer, every comedo or pimple exciting alarm. He developed a small epithelioma in the outer end of the right brow. In the dark, this lesion was struck against the end of a plank, the wound healed and there was no further sign of disease there. Some months later the right pre-auricular gland was found enlarged to the size of a bean.

Growth continued, a wrong diagnosis was made, operation and treatment too late and irregular, the patient died of involvement of the whole right face. The original lesion would seem to have sent metastasis to the gland before its own accidental destruction.

This case and the eldest daughter of the patient seem to indicate auto-suggestion. The daughter married late. At the age of about forty, her one child was two or three years old.

Following a blow from child's head a rapidly growing carcinoma of the right breast developed. This breast, later the other and various body recurrences were treated by radical surgery—in another city. The patient died in a little over two years. Following the death from cancer of her two maternal aunts and her father she had become obsessed with the dread of this disease and frequently consulted me about a little adenoma in the lower edge of the right breast. When I, later, saw and diagnosed the malignant disease of this breast, I was surprised to find the adenoma *not* involved in the general mammary infiltration.

A young unmarried woman had, since childhood, this dread and expectation of cancer. The radical operation was done for adenocarcinoma of the left breast. I later removed recurrent nodules from the skin of chest-wall, which had appeared in spite of X-ray treatment. Though she lived over a year after the last operation, there was no further appearance of disease in this region, but following pain in the lower third of the left femur, of some weeks duration, the bone broke while she was hurriedly walking. There was no extension of disease around the ununited fracture, but osteo-carcinomatous nodes formed on the frontal and parietal bones.

Her death, from brain pressure symptoms, indicated similar involvement of the inner table of the skull. This case almost shows auto-suggestion and is an example of remarkable metastasis.

Heredity in cancer is not proven, the proper explanation of familial cases being inherited tissues susceptibility to the disease. Contagion to another is extremely doubtful, so much so that workers in this disease have no fear of it.

Auto-contagion, or trans-plantation, however, is a well known occurrence, just as certain skin diseases are auto-infectious.

One of the most harmful errors ever disseminated by the press was that the late Dr. Bull of New York was a cancer specialist and contracted the disease in his practice. He was a general surgeon, took cancer cases as a part of his work and died of cancer just as a layman might.

In spite of non-heredity and non-contagion, curious cases occur—as the following. A man whose prepuce was always phimotic died of cancer of the penis a year or two after his wife died of cancer of the uterus, and his father had long before died of cancer of the penis.

A man died in the cancer age of epithelioma developing in a deep dimple of the chin. His son had a similar dimple, developed epithelioma there at about twenty-five years of age, died of the disease in a few years. This was the first case in the South treated by X-rays and at one time seemed cured.

Melanoma originating in a five cent sized brown spot on an elderly man's foot. Growth, metastasis and death. A physician who saw this case had on his foot, on the inner side of instep, a small brown pigmented lesion, and was alarmed about it for years—then forgot. After fifteen years this lesion is now a faint pinkish brown, is fading away. It has always been carefully protected from irritation.

A dark mole on a man's arm became melanotic, metastasised, caused death. A similar lesion, irritated by the suspender, on another man's shoulder, grew, had rather in-complete treatment, gave no further trouble.

Sarcoma, being of the embryonic type, rapidly growing yet less resistant in many cases, has furnished the most of the cures reported as following various new treatments. Its behavior is often freakish and startling.

A young man developed an osteo-sarcoma of the inner surface of the right ilium. The tumor was exposed by incision, pronounced inoperable, the wound closed without drainage. I was asked to give X-ray treatments, found the man very weak, running septic temperature and constantly soaked with perspiration. He could not take treatment. During the absence of his surgeon I found an abscess under the wound line, incised, and evacuated about half-pint of pus. Drainage was kept up, the patient returned to his home, got well, weight back to 165 pounds. This case was cured by the infection.

I have seen a small epithelioma, the point of infection in a severe case of erysipelas, heal during the acute disease and recur after it had passed. My belief is that the good, though temporary, effect in this case and the effects of Coley's fluid, Trypsin and perhaps serums has been due chiefly to the rise in temperature.

If a malignant growth could be kept constantly at high temperature it might be controlled. Heat does reduce the inoculability, or vitality of some experimental tumors.

A prisoner under sentence of death rarely dies of disease. A patient with cancer almost never has any other illness. One exception was the case of a man, aged seventy-eight, who had been almost cured of a large papillomatous epithelioma of the chest by X-rays, but with less help to a similar lesion beneath the right axilla,—died, at the end of a four weeks' seige, of dysentery.

The pain of cancer is peculiar. In the early stages there may be just a little itching, or no subjective symptoms, in an epithelioma. Carcinoma is frequently permitted to reach a hopeless stage before the patient seeks help, because there has been no pain. For example "just noticed a little hard lump in the breast," or "have been bleeding from uterus, but thought it just the change of life." A stomach case believes it indigestion.

When pain does occur it is of darting, shooting, needle or knife thrust character, at irregular intervals. This may occur in even a small lesion, and is very characteristic of carcinoma. Later, of course may occur ordinary ulceration, or necrosis pains or those from pressure.

The occurrence of pain so late in the disease is one of its most fiendishly malignant features, because the patient feels that the early absence of pain indicates there is nothing serious.

The principles of treatment have improved but little. We have learned to do more thorough and extensive work and to urge early intervention, have tried to educate our patients, but are still groping for a real cure of far-gone cases. Imitations of procedures for the cure of other diseases are being followed, empiric experiments are being made. The infective or causative agent is still, however, unknown, adding vastly to our difficulties, depressing in the futility of much of our labor, but the fight must be continued until this great opprobrium medicorum can be removed. Even though we may know cause and cure we shall still have human ignorance, procrastination, superstition, charlatanism and general imbecility to combat.

A certain number of people must follow their destiny, or commit suicide even if indirectly. For these physicians cannot be held responsible and their fate need not rest on our conscience. If we can rescue those who try we shall have accomplished all that can be expected of us and education should constantly increase the number—as our knowledge and capability improve.

503-4 The Grand

